# The Effects of Extracellular Vesicles From Cancer Cells in Acidosis or ER Stress to Endothelial Cells: Angiogenesis and Endothelial Activation

**DOI:** 10.1111/boc.70085

**Published:** 2026-10-07

**Authors:** Ana Sayuri Yamagata, Ramon H. G. Teles, Nicolas J. Villarinho, Ana Carolina Lima Ralph, Nicolly C. dos Santos, Kevin Perera, Gisela R. Terçarioli, Amanda Ikegami, Anayara Ristow, Daniel Pecoraro Demarque, Jeremy Newton, Luiz E. da Silva, Edmond W. K. Young, Ruy G. Jaeger, Vanessa Morais Freitas

**Affiliations:** ^1^ Department of Cell Biology and Developmental Biology Institute of Biomedical Sciences University of São Paulo São Paulo São Paulo Brazil; ^2^ New Approaches in Radiotherapy Centre for Research in Molecular Medicine and Chronic Diseases (CiMUS), Santiago de Compostela University of Santiago de Compostela A Coruña Spain; ^3^ Faculdade de Farmácia Faculdade Estácio Carapicuíba Brazil; ^4^ Hospital das Clínicas da Faculdade de Medicina da USP HCFMUSP Universidade de São Paulo São Paulo São Paulo Brazil; ^5^ Department of Mechanical & Industrial Engineering University of Toronto Toronto Ontario Canada; ^6^ International Research Center A.C. Camargo Cancer Center São Paulo Brazil; ^7^ Laboratório de Farmacognosia Faculdade de Ciências Farmacêuticas Universidade de São Paulo São Paulo São Paulo Brazil; ^8^ Institute of Biomedical Engineering University of Toronto Toronto Ontario Canada; ^9^ Department of Materials Science & Engineering University of Toronto Toronto Ontario Canada

**Keywords:** acidosis, cancer, endoplasmic reticulum stress, endothelial cells, extracellular vesicles

## Abstract

Tumor‐derived extracellular vesicles (EVs) facilitate metastatic progression by transferring bioactive cargo to recipient cells, thereby increasing endothelial permeability and promoting angiogenesis. The harsh conditions of the tumor microenvironment, such as extracellular acidosis, can alter the content of EVs, leading to changes in recipient cells, including endoplasmic reticulum (ER) stress. This study aimed to investigate whether EVs released by tumor cells adapted to acidosis or ER stress can alter endothelial functional properties. ER stress was induced in MDA‐MB‐231 breast cancer cells with tunicamycin, and adaptation to acidosis was achieved by culturing at pH 6.5 for 30 days. Large extracellular vesicles (LEVs) in these conditions were isolated by centrifugation. LEVs were characterized by nanoparticle tracking analysis (NTA), immunogold, Western blot, and high‐performance liquid chromatography (HPLC). ER stress and autophagy markers were quantified by Western blot; cancer cells' adhesion to endothelial cells was assessed by adhesion assay; barrier permeability by dextran diffusion in an organ‐on‐a‐chip model; migration by wound healing assay, and angiogenesis by tube formation assay. Tunicamycin was not detected in LEV from tunicamycin‐treated cells (LEV T). LEV T increased the migration of endothelial cells, while not inducing ER stress, nor altering adhesion, angiogenesis, or permeability. Acidosis‐adapted cells did not have greater UPR activation, but their LEVs (LEV 6.5) induced ER stress, reduced adhesion and angiogenesis, and increased permeability and migration. These findings indicate that LEVs from acidosis‐adapted tumor cells reprogram endothelial physiology, supporting a potential role in metastatic progression.

## Graphical abstract

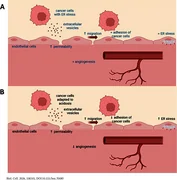

## 1 Introduction

Breast cancer (BC) is the most prevalent and has the highest mortality rate in women (Bray et al. 2024). Among its subtypes, the triple‐negative breast cancer (TNBC), which lacks expression of receptors for estrogen (ER), human epidermal growth factor (HER2), and progesterone (PR), accounts for 20% of BC cases and has higher malignancy and risk of metastasis to the brain, liver, and lungs (Kumar and Aggarwal 2016). Although 91% of TNBC patients survive within 5 years of diagnosis, the survival rate drops to 31% when there is metastasis to a distant organ (American Cancer Society 2024).

Metastasis establishment occurs through a well‐characterized process known as the metastatic cascade. In this context, tumor cells detach from the vascularized solid tumor, intravasate into blood vessels, engage in unstable attachment followed by firm adhesion to the endothelium, and extravasate to a secondary organ, leading to the development of a vascularized secondary tumor (Fares et al. 2020). Primary tumors are known to precondition secondary organ microenvironments by secreting soluble factors and releasing extracellular vesicles (EVs), which together may promote metastatic colonization (Chin and Wang 2016; Kaplan et al. 2005; Psaila and Lyden 2009).

In BC cases, tumor‐derived EVs from female patients have been implicated in changes of endothelial cell functioning, such as cellular migration and angiogenesis (Garcia‐Hernandez et al. 2022). The content of EVs can be influenced by cellular conditions, including adaptation to acidosis (Boussadia et al. 2018). However, the influence of tumor microenvironmental conditions, specifically acidosis, on the cargo of extracellular vesicles is still poorly understood. We aimed to study the effects of EVs derived from ER‐stressed breast tumor cells on autophagy, angiogenesis, and endothelial barrier integrity, which are implicated processes in metastasis establishment and progression.  By comparing LEVs released from tunicamycin‐treated and acidosis‐adapted tumor cells, we uncover distinct effects on endothelial ER stress, adhesion, permeability, angiogenesis, and migration. Our findings reveal that acidosis‐adapted tumor cells generate LEVs that actively reprogram endothelial physiology, providing a mechanistic link between tumor metabolic stress and the establishment of a pro‐metastatic vascular niche.

## 2 Results

### 2.1 Tunicamycin at a Low Concentration Induced ER Stress in MDA‐MB‐231 Cells

To induce ER stress, tunicamycin (Tm) was used. Mimicking the conditions of preparation of conditioned medium for EV extraction, the cells were treated with Tm for 24 h, washed three times in PBS, and incubated in serum‐free medium to reduce the contamination of EVs from FBS. First, the cellular viability of MDA‐MB‐231 was evaluated by MTT assay after incubation with different concentrations of Tm for 24 h (Figure 1A) and in serum‐free medium for another 24 h (Figure 1B). 500 ng/mL (0,5 µg/mL) was included based on other studies using similar concentrations of Tm to obtain EVs from cells with ER stress (Liu et al. 2019; Yao et al. 2020). However, lower concentrations were added because incubation in serum‐free media could further impact cell viability. After the first 24 h of incubation, a slight but statistically significant decrease in cell viability was observed from 1 to 500 ng/mL of Tm, while after another 24 h in serum‐free medium, cell recovery was observed from 1 to 5 nm/mL of Tm, returning to basal levels, except for the concentrations of 50 and 500 ng/mL. This indicates that cells preincubated with the lowest concentrations experienced ER stress but were still able to recover.

**Figure 1:**
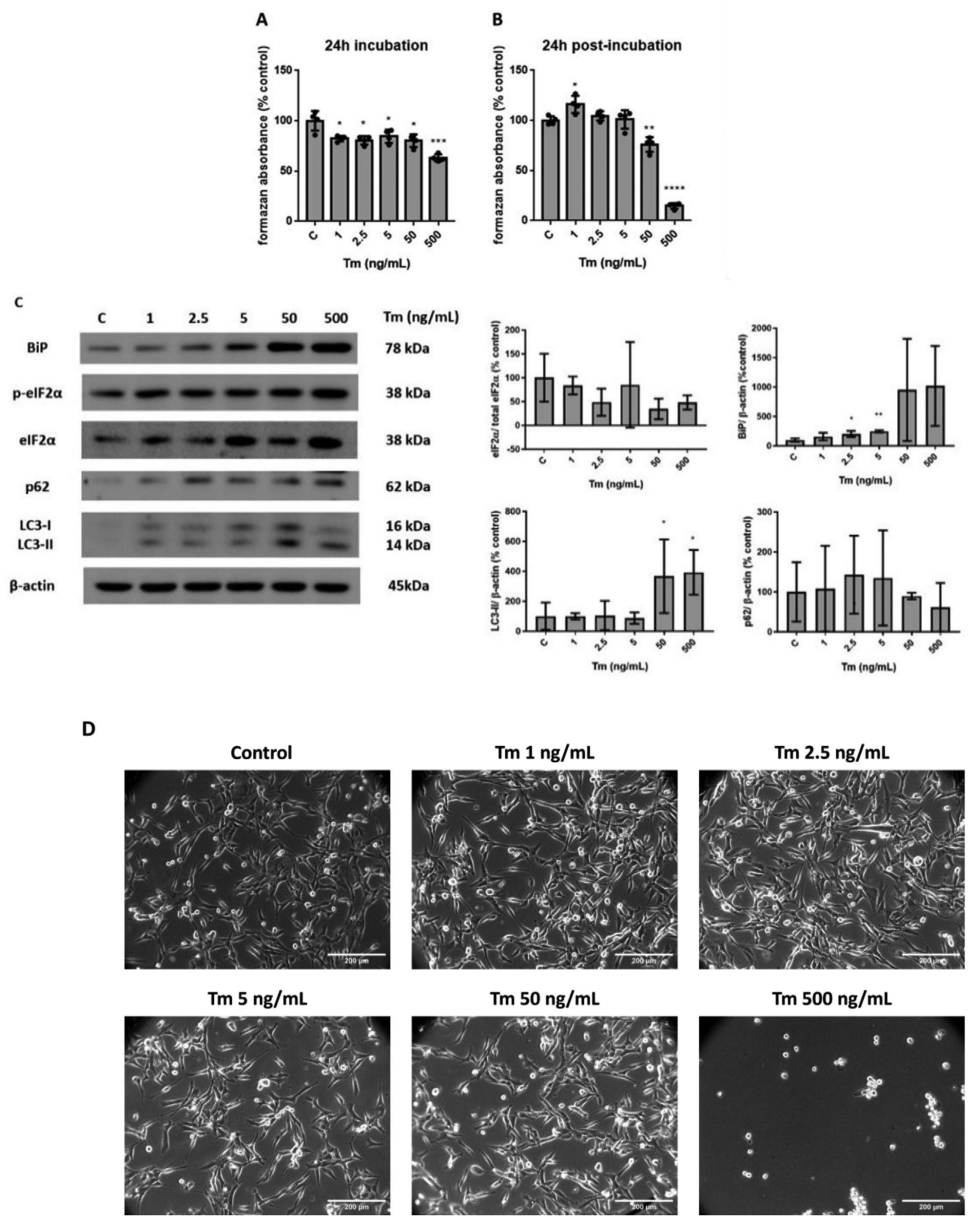
TNBC cells were exposed to different concentrations of Tunicamycin. (A) MDA‐MB‐231 cells were incubated for 24 h with increasing concentrations of Tm. (B) MDA‐MB‐231 cells were re‐evaluated in Tm‐ and serum‐free media after another 24 h. (C) The protein makers p‐eIF2α/ eIF2α and BiP were assessed by Western blot. (D) Light microscopy images in contrast and phase 24 h after the end of drug incubation. Scale bar: 200 µm. Data was displayed as mean ± SD. * *p* < 0.05; ** *p* < 0.01; *** *p* < 0.001; **** *p* < 0.0001 compared to the control group (C).

To confirm the ER‐stress in the cells incubated with Tm, after 24 h, p‐eIF2α/eIF2α and BiP were quantified, while LC3 and p62 were used to evaluate autophagy (Figure 1C). An increase in the amount of BiP was observed in the cells treated with 2.5 and 5 ng/mL of Tm, which strongly suggests that ER stress has occurred. The LC3‐II level was only increased at 500 ng/mL, but was not accompanied by a reduction in p62, indicating the absence of autophagy (Klionsky et al. 2021).

Cell morphology was assessed by light microscopy. Those incubated with 500 ng/mL acquired a rounded shape characteristic of the apoptotic process, while the other concentrations did not show a great impact on the confluence or morphology, compared to the control (Figure 1D), except for the concentration of 2.5 ng/mL, which seemed to affect the cellular elongation by a diffuse membrane protrusion, acquiring a pronounced spindle‐like morphology. In summary, low‐dose tunicamycin induced ER stress in MDA‐MB‐231 cells without significant alterations in viability and morphology, whereas higher doses led to metabolic dysfunction and apoptotic features.

Considering these results, we further tested whether 2.5 ng/mL of tunicamycin would dilate the rough ER as a sign of ER stress. The treated cells presented enlarged ER, as observed in transmission electron microscopy (Figure 2A). Therefore, 2.5 ng/mL Tm was chosen to activate ER stress to obtain EVs.

**Figure 2:**
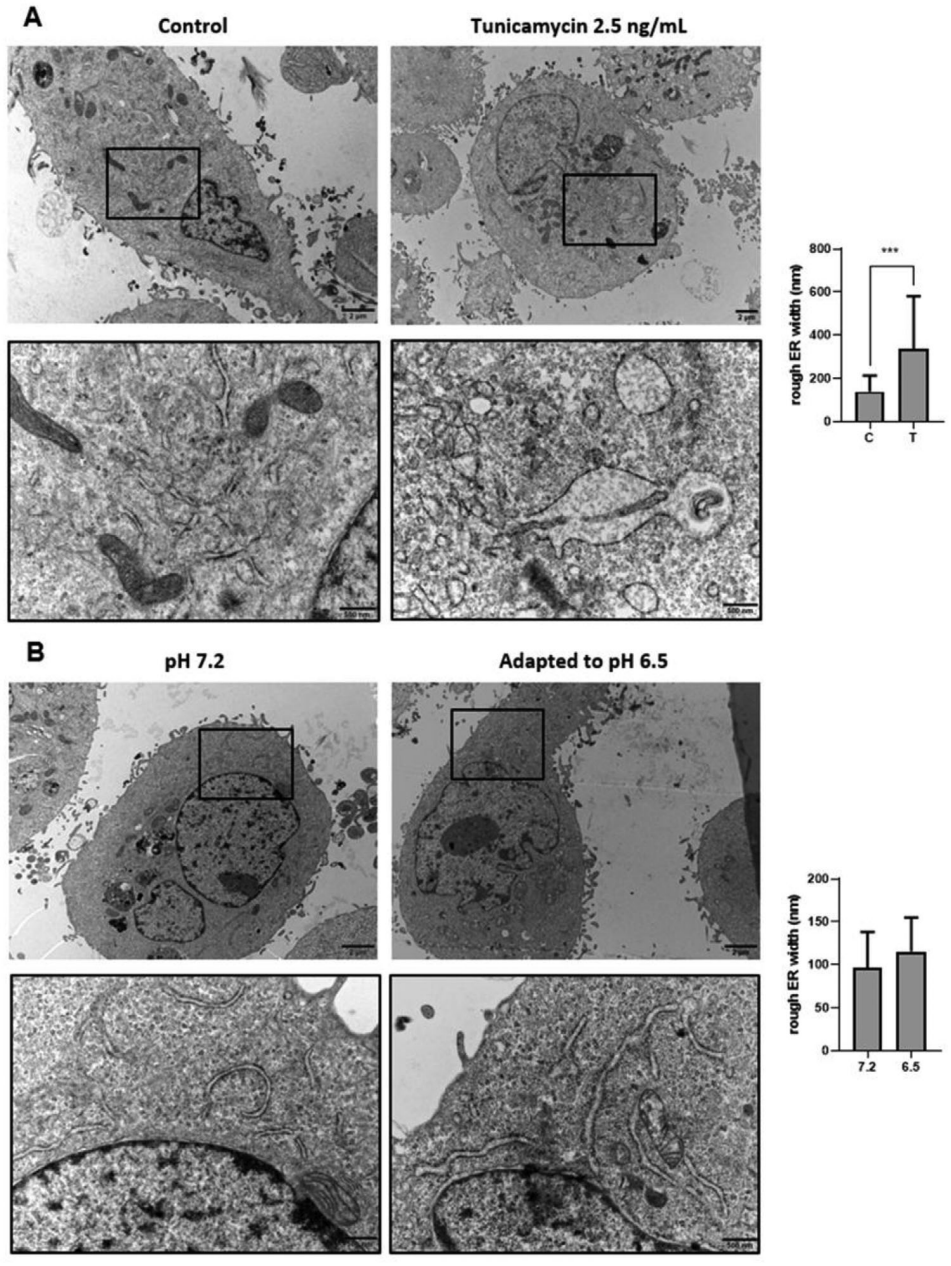
Rough endoplasmic reticulum width measured in micrographs of transmission electron microscopy. (A) MDA‐MB‐231 treated with 2.5 ng/mL tunicamycin and control. (B) MDA‐MB‐231 cultured at pH 7.2 as a control or adapted to pH 6.5. Data was displayed as mean ± SD of the measurements taken within the same sample. *** *p* < 0.001.

Additionally, considering that acute acidosis (3 h) can cause ER stress (Aoyama et al. 2005), the cells were cultured at pH 6.5 or pH 7.2 for one month; however, no significant difference in ER width was found in acidosis‐adapted cells (Figure 2B). Low‐dose tunicamycin induced ER dilation indicative of mild ER stress, whereas long‐term acidosis adaptation did not alter ER morphology.

### 2.2 Characterization of EVs

The large EVs (LEV) samples, precipitated at 15,000×g, were characterized by nanoparticle tracking analysis (NTA) to observe the particle size and concentration profile. The LEV distribution obtained showed a concentration peak around 165 nm for both control and ER‐stressed TNBC cells (Figure 3A).

**Figure 3:**
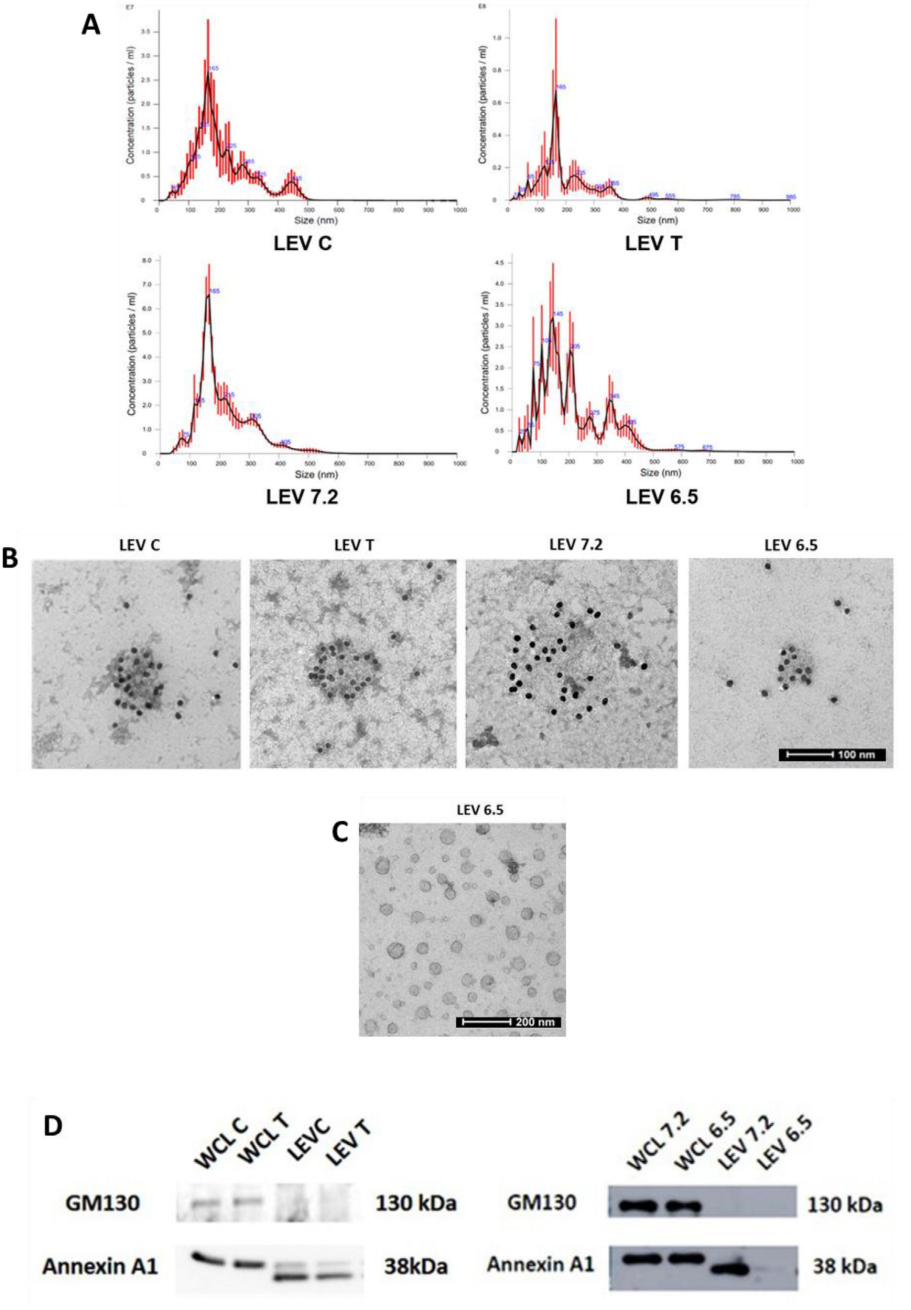
Characterization of MDA‐MB‐231 large extracellular vesicles (LEVs) with or without tunicamycin (Tm) treatment under control (pH 7.2) and acidosis‐adapted (pH 6.5) conditions. (A) Nanoparticle tracking analysis (NTA) of LEVs derived from MDA‐MB‐231 cells treated with tunicamycin (T) or untreated controls (C), cultured under control pH (7.2) or adapted to acidic pH (6.5). Data represent the mean ± standard error of the mean (SEM) from five readings of the same sample (red bars). The accompanying table displays the mean and mode particle diameters for each condition. (B) Transmission electron microscopy (TEM) of LEVs labeled for Annexin A1 by immunogold. Representative images are shown for LEV C, LEV T, LEV 7.2, and LEV 6.5 samples. (C) Example of Annexin A1‐negative LEVs observed in the LEV 6.5 condition. (D) Western blot analysis of whole‐cell lysates (WCL) and LEVs showing the presence of the microvesicle marker Annexin A1 and absence of the Golgi marker GM130 (negative control).

To confirm the proteic profile of the isolated LEV following the differential centrifugation methodology, they were stained for annexin A1 (ANXA1), a protein enriched in microvesicles (Jeppesen et al. 2019), and visualized by transmission electron microscopy (Figure 3B). The LEV 7.2, LEV 6.5, LEV C, and LEV T samples presented annexin A1‐positive vesicles with an average size of 179.4 ± 5.3, 179.4 ± 5.3, 140.5 ± 41.0, and 199.3 ± 39.5 nm, respectively. Additionally, a large number of annexin A1–negative EVs (*n* = 106), with an average diameter of 59 nm, were detected in a localized region of the LEV 6.5 preparation, suggesting the presence of a distinct small‐EV subpopulation and highlighting vesicular heterogeneity under acidosis‐adapted conditions (Figure 3C). It should be noted that the immunogold TEM preparation protocol includes fixation with paraformaldehyde, dehydration, and drying steps that may affect EV morphology. Therefore, these images were used to assess ANXA1 localization and not for accurate vesicle size determination, which was evaluated by NTA.

The protein profile of LEVs was analyzed by western blot, detecting the ANXA1‐cleaved form only in the vesicles, while evidencing the full‐length form in the cell lysates. Noteworthy, in the LEV 6.5, the ANXA1 band was exceptionally weak but still present, confirmed by the assay repetitions (data not shown). This is the first time that this protein has been analyzed and observed in LEV from cells adapted to acidosis. Furthermore, GM130, a resident protein of the Golgi complex, was observed in the cells but absent in EVs, meaning that the EV samples were free of lysed cell fragments (Figure 3D). It is important to mention that CD9, a positive control for the presence of small vesicles, was not detected in LEV 6.5 nor LEV T (Supporting Information), indicating the possibility of changes in the protein markers.

To detect whether Tm was being transported to endothelial cells by LEV T, we analyzed the EVs content by high‐performance liquid chromatography (HPLC). Tm was first analyzed as a standard, which contains the 4 variants of the drug, A, B, C and D (Figure S1A). Four peaks were observed in greater quantity, which should correspond to the 4 variants. In LEV T, these same four peaks were not detected, apart from a peak with a retention time of 38.569 min (Figure S1B). Since this peak in the LEV T sample has a similar value to the peak at time 38.743 min in the tunicamycin drug standard, the UV absorption spectrum graphs of these peaks were compared (Figure S1C,D). The UV spectrum curves were distinct, leading to the conclusion that these peaks do not correspond to the same molecule. The peak at 38.569 min in LEV T was not detected in the LEV C sample (Figure S1E). Thus, we did not find evidence of tunicamycin in the isolated LEV that would be carried over to the recipient cells.

### 2.3 Large Extracellular Vesicles (LEV) Do Not Alter Endothelial Cell Metabolism

Human umbilical vein endothelial cells (HUVEC) were treated with LEVs Tm, 7.2, 6.5, and control at a concentration of 0.5 µg protein/mL for 24 h. Cell metabolism was assessed by MTT assay (Figure 4). No significant differences were found between conditions, suggesting that the concentration used was not toxic to the cells and was appropriate for subsequent experiments.

**Figure 4:**
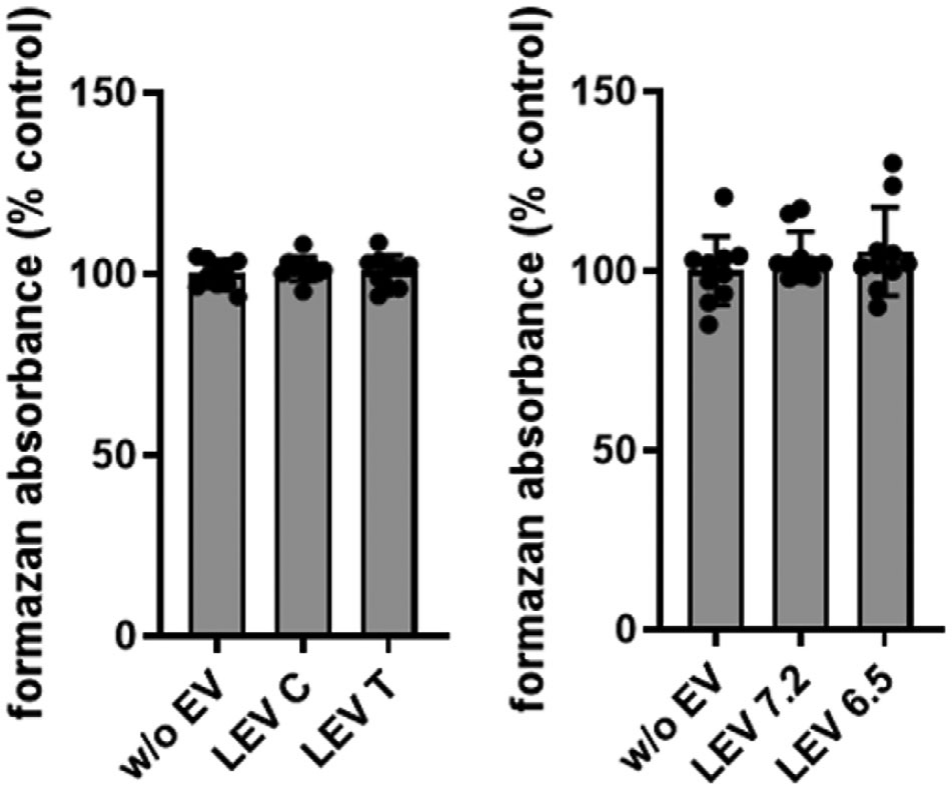
Assessment of viability of HUVEC treated with LEV. MTT assay in HUVEC treated for 24 h with LEV C, LEV T, LEV 7.2, and LEV 6.5 from MDA‐MB‐231. After LEV treatment, HUVECs were incubated with MTT 0.5 mg/mL for 3 h. The formazan metabolite was solubilized in DMSO, and the absorbance was measured at 540 nm. Data from three independent replicates was displayed as mean ± SEM.

### 2.4 LEV T and LEV 6.5 Influence Metastasis Steps Related to Endothelial Cells

The major steps of metastasis in which endothelial cells participate are adhesion, increase in permeability, for extravasation, and angiogenesis, in this order. Next, it was tested whether LEV T or LEV 6.5 would alter the adhesion of MDA‐MB‐231 to HUVEC or the extracellular presence of VCAM‐1 in immunofluorescence. VCAM‐1 may be expressed by both endothelial and tumor cells. In the endothelium, VCAM‐1 has been reported to interact with β1 integrins on tumor cells, promoting firm tumor cell adhesion to the vascular wall and facilitating extravasation and metastatic spread (Läubli and Borsig 2019). LEV 7.2 increased MDA‐MB‐231, while the same effect was not detected in LEV C (Figure 5A). This difference could not be explained. Neither LEV C nor LEV T vesicles altered the amount of adhered MDA‐MB‐231 (Figure 5A). In the LEV 6.5 condition, there were fewer adherent tumor cells compared to the treatment with LEV 7.2 (Figure 5B). Also, no difference was observed regarding VCAM‐1 labeling, indicating that another mechanism of action can be implied in this response (Figure 5A,B).

**Figure 5:**
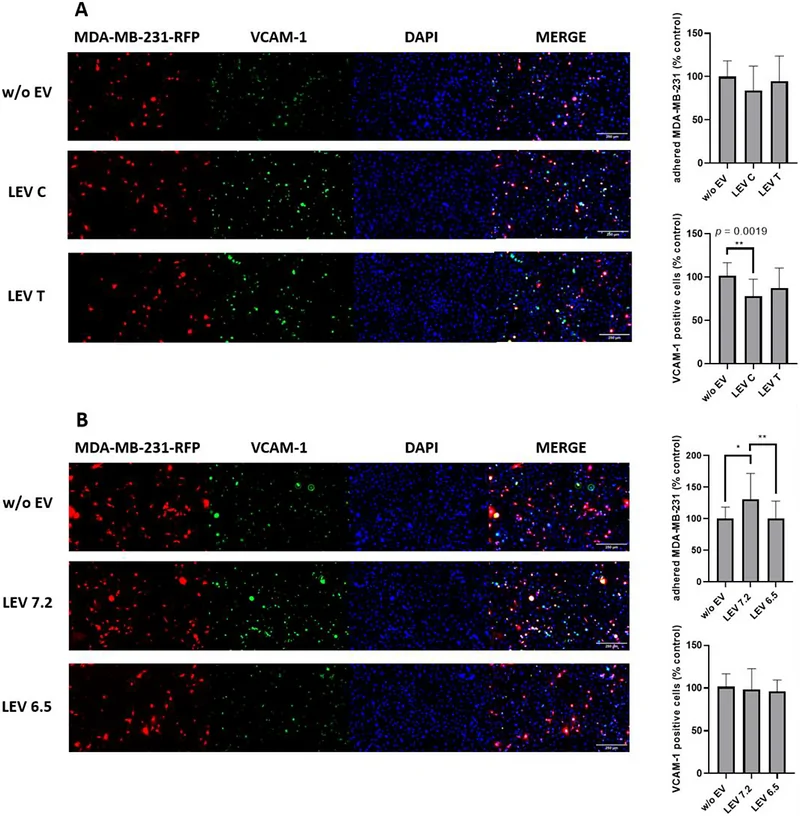
LEV 6.5 reduces MDA‐MB‐231 adhesion. Adhesion assay of MDA‐MB‐231‐RFP (expresses red fluorescent protein) on HUVECs pre‐incubated with LEV for 5 h. Cells were stained for VCAM‐1 (green), and nuclei were stained with DAPI (blue). Scale bar: 250 µm. Data from three independent experiments was displayed as mean ± SEM of the quantifications from five images from each coverslip, one coverslip for each condition, in three repetitions. * *p* < 0.05; ** *p* < 0.01. (A) HUVECs treated with LEV C and LEV T; (B) or LEV 7.2 and LEV 6.5.

Endothelial permeability in response to LEV was evaluated by a FITC‐dextran diffusion assay through an HUVEC monolayer seeded on collagen gel in an organ‐on‐a‐chip microfluidic device known as TANDEM (Mohan and Young 2021) that has previously been applied for studying pancreatic cancer (Mohan et al. 2024). First, the integrity of the endothelial barrier was verified by VE‐cadherin immunofluorescence. HUVECs seeded on the collagen gel chip were alive and formed cell‐cell junctions constituting a barrier (Figure 6A). Because the collagen surface was oblique, as detailed in the scheme of the microchip (Figure 11), the immunofluorescence micrographs were captured in Z‐stack. The shown image, which is a projection of the stacked images, gives support to the interpretation that a monolayer was formed rather than a multilayer. Medium with FITC‐dextran and treatment (TNF‐α or EVs) was injected into the same lateral channel where HUVECs were seeded, and the diffusion of dextran from the lateral channel to the central channel was monitored (Figure 6B). Treatment with TNF‐α 100 ng/mL, as a positive control, increased endothelial permeability relative to untreated (Figure 6B,C), showing that the device and protocol were successful in assessing permeability.

**Figure 6:**
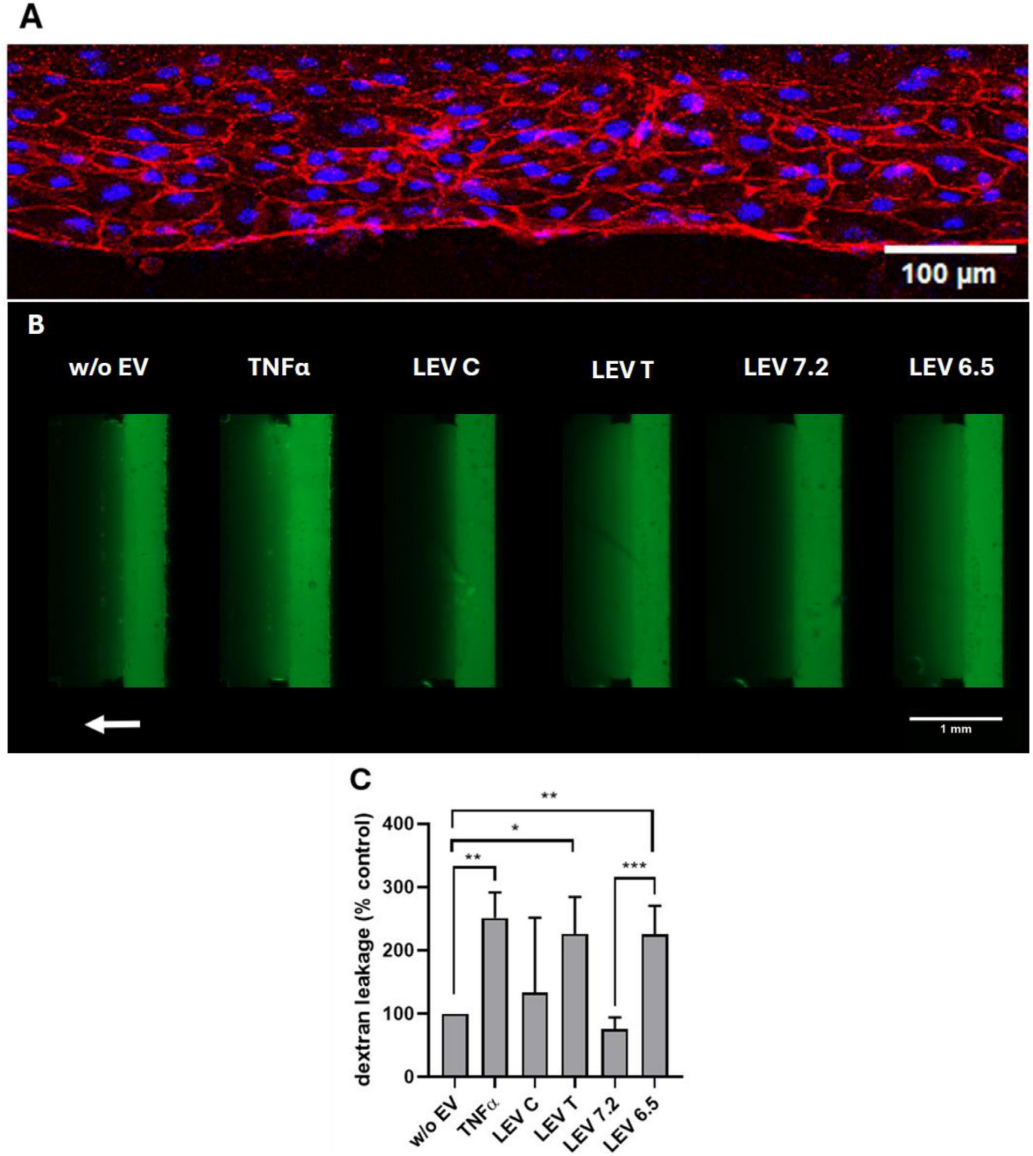
LEV T and LEV 6.5 increase endothelial permeability. Permeability assay on the modified TANDEM chip. (A) VE‐cadherin staining of HUVEC seeded in collagen gel within the chip. VE‐cadherin in red and Hoechst‐stained nuclei in blue. Scale bar: 100 µm. (B) Images of the central and lateral channels of the different conditions 60 min after treatment injection. The arrow indicates the direction of diffusion of dextran‐FITC from the right channel to the central channel, through a layer of HUVEC. Scale bar: 1 mm. (C) Analysis of dextran‐FITC diffusion through the endothelial layer estimated by the fluorescence intensity in the channels. Data from three independent experiments was displayed as mean ± SD. * *p* < 0.05; ** *p* < 0.01; *** *p* < 0.001.

No changes in endothelial permeability were observed following treatment with LEV C compared with untreated cells (w/o EV), and permeability remained similar between the LEV C and LEV T groups. Nevertheless, LEV T significantly increased permeability relative to the w/o EV control. Likewise, LEVs isolated from cells cultured at pH 6.5 promoted greater FITC‐dextran extravasation than LEVs derived from cells maintained at pH 7.2, as well as the w/o EV control group (Figure 6B,C). Overall, LEV T and LEV 6.5 increased permeability compared to the w/o EV control.

After the increase in leakage through the endothelial barrier, angiogenesis is the following event in metastasis. To assess the impact of LEVs on angiogenic behavior, wound healing and tube formation assays were conducted using HUVECs. Overall, control LEVs (C and 7.2) reduced HUVEC migration relative to w/o EV (Figure 7A,B, whereas LEVs derived from tunicamycin‐treated cells (T) or from acidosis‐adapted conditions (6.5) significantly enhanced migration compared to their respective controls, LEV C and LEV 7.2, rescuing the effect. In contrast, in the tube formation assay, LEV 6.5 slightly decreased the number of loops formed in HUVEC networks, while no significant differences were observed among the other conditions (Figure 8A,B). Therefore, the data indicates that LEV 6.5 may inhibit angiogenesis.

**Figure 7:**
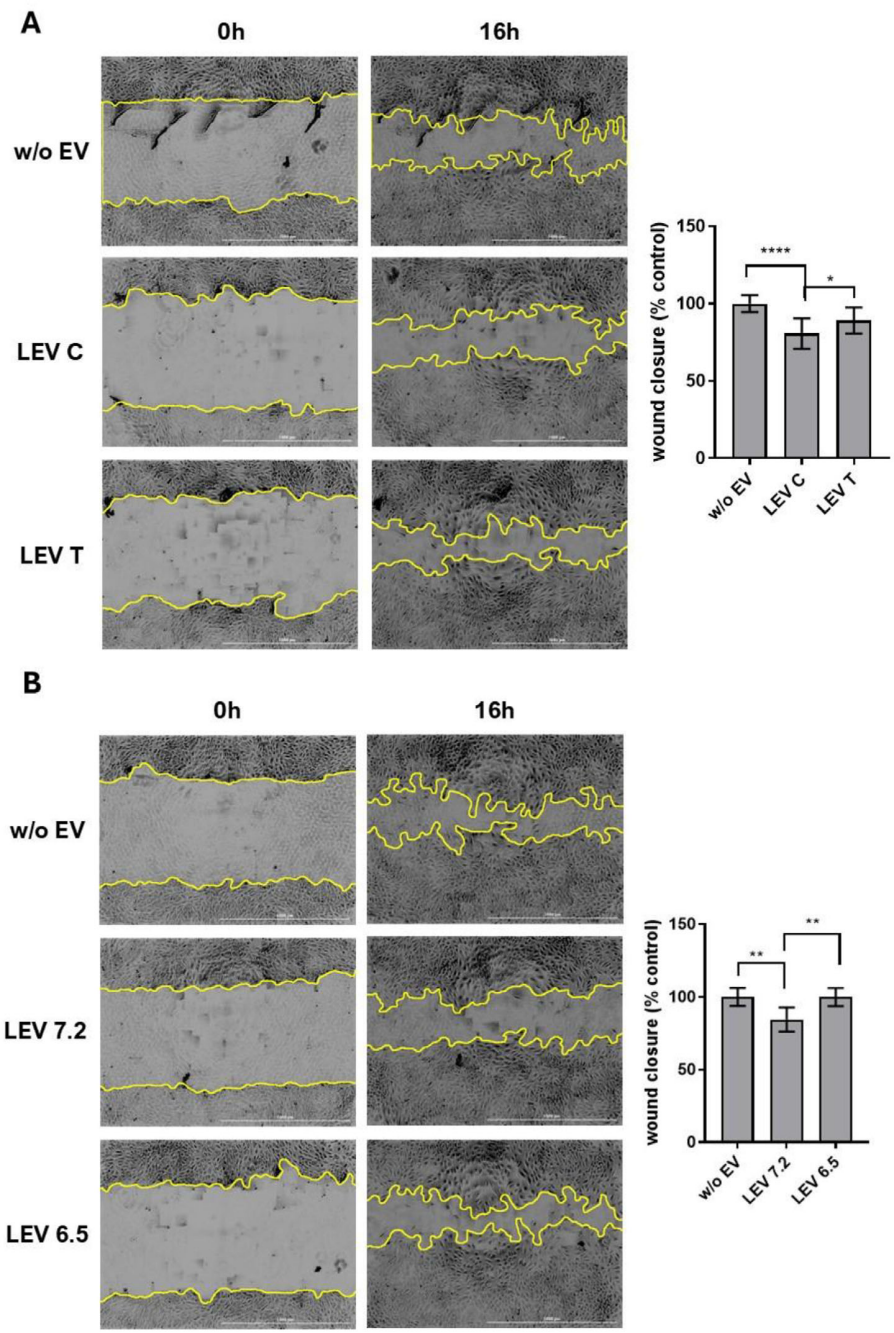
LEV T and LEV 6.5 increase HUVEC migration compared to control LEVs. Wound healing assay of HUVEC treated with (A). LEV C and LEV T, (B) LEV 7.2 and LEV 6.5. HUVEC were incubated with vesicles for 24 h, treated with mitomycin 5 µg/mL for 2 h, scratched, and then incubated with vesicles again during wound closure. Scale bar: 1 mm. Data from three independent experiments was displayed as mean ± SD. * *p* < 0.05; ** *p* < 0.01; **** *p* < 0.0001.

**Figure 8:**
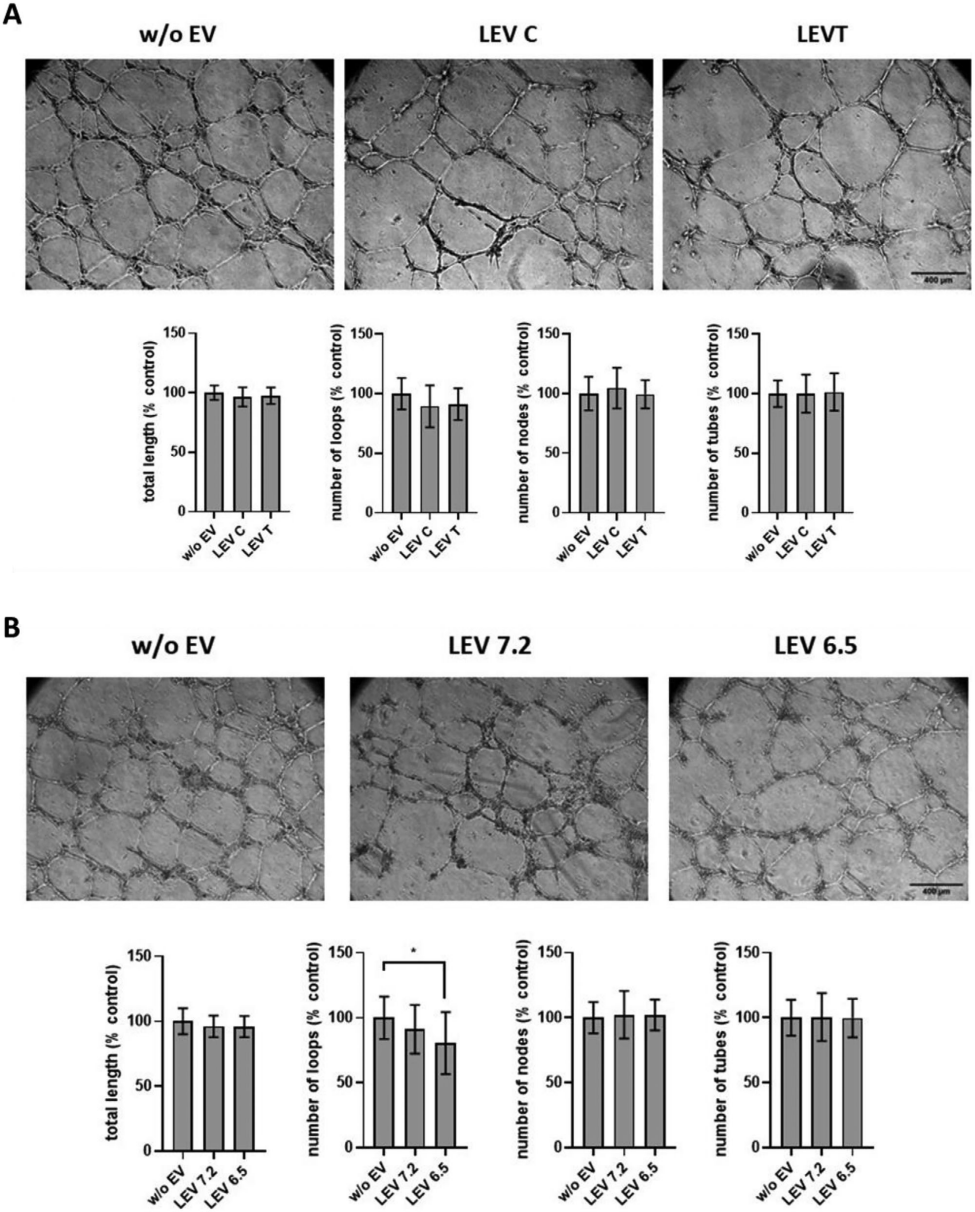
LEV 6.5 slightly reduces tube formation. Tube formation assay of HUVEC treated with (A). LEV C and LEV T, (B) LEV 7.2 and LEV 6.5. HUVEC in medium with LEV were plated on matrigel‐lined wells, and tube‐like structures were observed after 16 h. Data from three independent experiments was displayed as mean ± SD. * *p* < 0.05.

### 2.5 LEV 6.5 Induces ER Stress in Endothelial Cells

To investigate a possible mechanism of action, the potential modulation of ER stress and autophagy by LEVs in HUVECs was assessed. HUVEC treated with Tm 0.5 µg/mL was used as positive control. As expected, HUVECs exposed to Tm exhibited elevated levels of the ER chaperone BiP, confirming the effectiveness of the positive control for ER stress induction (Figure 9A,B). LEV C and LEV 7.2 did not cause significant changes in the expression of ER stress‐ or autophagy‐related proteins compared to untreated cells. However, exposure to LEV 6.5 resulted in a modest increase in the ratio of phosphorylated eIF2α relative to total eIF2α, suggesting a mild activation of the ER stress response.

**Figure 9:**
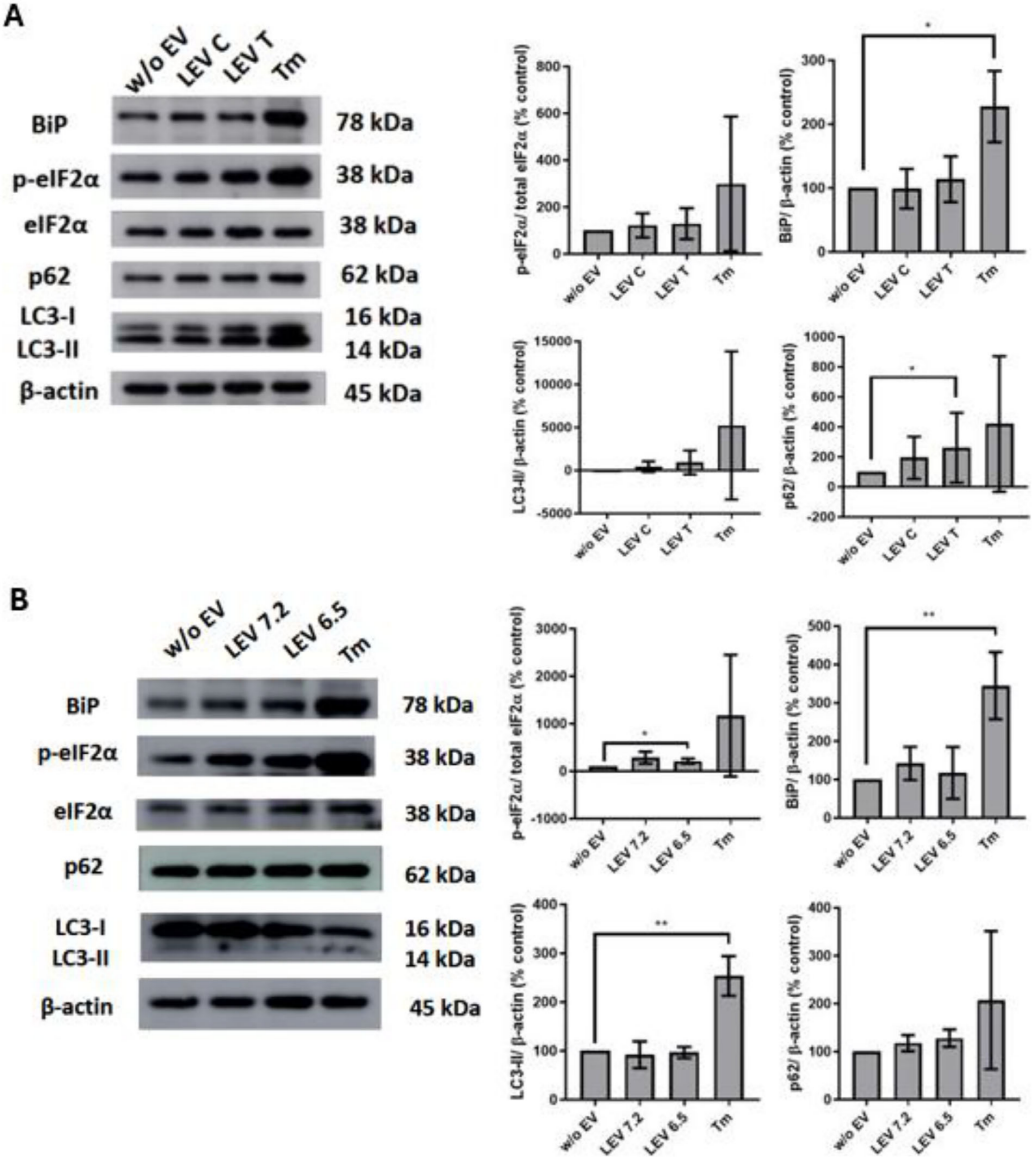
LEV 6.5 activates UPR by p‐eIF2α. HUVECs were treated with LEV C, LEV T, or tunicamycin (Tm) at 0.5 µg/mL for 24 h, following western blot assays to detect BiP, p‐eIF2α, total eIF2α, p62, LC3‐I and II, and β‐actin. Results from three independent replicates. (A) HUVECs treated with LEV C and LEV T; (B) or LEV 7.2 and LEV 6.5. Data from three independent experiments was displayed as mean ± SD. * *p* < 0.05; ** *p* < 0.01.

## 3 Discussion

EVs alter the microenvironment, favoring cancer cell colonization before their arrival and contributing to the establishment of a premetastatic niche (PMN) (dos Anjos Pultz et al. 2017). Since environmental conditions such as acidosis and hypoxia alter EV protein cargo and properties (Boussadia et al. 2018), this study investigated how adaptation to acidosis and ER stress influence the effects of tumor‐derived EVs on endothelial cells.

The vesicle size profile observed by NTA was similar to that of EVs isolated from plasma of breast cancer patients (Ramirez‐Rícardo et al. 2020). Based on particle sizes, LEV samples contain LEVs and small EVs (Dang et al. 2020; Doyle and Wang 2019). EV samples were enriched in annexin A1 (ANXA1), a marker of microvesicles/ectosomes (Jeppesen et al. 2019), except for LEV 6.5, whose band was weak but present. In the membrane, ANXA1 can be cleaved by metalloproteases (Boudhraa et al. 2016) and, when attached to the inner face of the membrane, it is flipped by flippases and then ejected into ectosomes/microvesicles by evagination (Boudhraa et al. 2016). However, ANXA1 controls intracellular Ca^2+^ (Frey et al. 1999), which is needed for adaptation to extracellular acidosis by exocytosis of H^+^‐containing vesicles via Ca^2+^‐dependent signaling (Shuichi Tsuru and Schwartz 1998). This explains the faint band of this protein in LEV 6.5. This is the first time that LEVs derived from acidosis‐adapted cells are characterized for expressing ANXA1, as EVs from this condition had only been analyzed in the literature for small EVs marker proteins, such as CD81, Alix, and TGS101 (Andreucci et al. 2022; Boussadia et al. 2018). In fact, by immunogold observed in transmission electron microscopy, EVs positive for annexin A1 were found in all samples, in addition to EVs without this protein.

The size of the vesicles was within the expected range considering the NTA. Unlike the size averages obtained through NTA, the EVs found in the LEV 6.5 sample were smaller than those in LEV 7.2, although this analysis is only qualitative.

Previous studies have explored the immunomodulatory effects of EVs from tumor cells that have undergone endoplasmic reticulum stress (Liu et al. 2019; Yao et al. 2020), in addition to altering angiogenesis (Wang et al. 2022). Other articles support the idea that EVs from tumors with an acidic microenvironment contribute to metastasis (Andreucci et al. 2022; Boussadia et al. 2018). Our group found that acute acidosis increases EV release, but leads to a trend towards reduced LEV release (Ralph et al. 2020). Accordingly, we observed fewer LEVs in the Tunicamycin and 6.5 groups. Other groups have observed that chronic acidosis increases the release of small vesicles (Boussadia et al. 2018; Parolini et al. 2009). Tumor cells with greater metastatic potential have a greater increase in the release of EVs in response to adaptation to acidosis (Boussadia et al. 2018).

We observed that LEV 6.5 reduced the adhesion of MDA‐MB‐231‐RFP to HUVEC. Interestingly, Rauschner et al. also observed that incubation at acidic pH makes tumor cells less adhesive (Rauschner et al. 2021). Perhaps, LEV 6.5 would be enriched with factors responsible for this effect. On the other hand, Boussadia et al. reported increased expression of proteins related to cell‐cell adhesion in melanoma cells adapted to acidosis (Boussadia et al. 2018).

Additionally, LEV 6.5 increased endothelial permeability. Interestingly, our group observed loosening of cell‐cell junctions between cancer cells in acute acidosis (Ralph et al. 2020). This phenotype is also induced in HUVEC receiving EVs from acid‐adapted tumor cells. This finding corroborates those that small EVs from acidosis‐adapted melanoma cells stimulate the release of pro‐inflammatory cytokines IL‐1β, IL‐6, and TNF‐α from macrophages, which increase the permeability and trans‐endothelial migration of tumor cells in their interior (Andreucci et al. 2022). Similarly, HUVECs treated with LEVs from HUVECs with ER stress induced by thapsigargin release more IL‐6 (Osman et al. 2020). The method used in this study to assess endothelial permeability has advantages over the popularly used transwell method. This organ‐on‐a‐chip model is representative because the endothelial cells are attached to a type I collagen gel, which is abundant in the extracellular matrix, instead of a polyester layer, in which the cells can grow through the pores (Biegel and Pachter 1994). Furthermore, it is possible to observe a confluent monolayer of cells on the gel, in addition to verifying the integrity of the endothelial layer, as done in the VE‐cadherin immunofluorescence.

There was a reduction in migration of HUVEC treated with control EVs, although it was not possible to do a time‐lapse to assess other time points. This was not expected, as other articles have reported that EVs from pancreatic tumor cells increased the migration of normal pancreatic cells (Hinzman et al. 2022) and EVs from plasma of breast cancer patients increased the migration of endothelial cells (Garcia‐Hernandez et al. 2022). In contrast, LEV 6.5 and LEV T increased migration, compared to control EVs. In agreement, acute and chronic acidosis increase tumor cell migration (Boussadia et al. 2018; Chen et al. 2022; Riemann et al. 2016). This finding was corroborated by the enrichment of proteins related to these cellular functions, in addition to cell‐cell adhesion as well as induction of migration in melanoma cells by exosomes produced by cells adapted to acidosis (Boussadia et al. 2018).

It is possible that LEV 6.5 is prone to decrease angiogenesis. Inhibition of angiogenesis was also observed when endothelial cells were incubated at pH 6.5, with a reduction in the number of tubes formed, an increase in VEGF secretion, and a reduction in IL‐8 (Huang et al. 2017). It is known that VEGFA influences the number and length of tubules (Merks et al. 2006), and the size of loops can be modulated by the degree of diffusion of morphogens, such as VEGF (Merks et al. 2006). However, to date, it is not known what specifically regulates the number of loops or what part of angiogenesis this represents. However, the lower number of loops together with the no change in the number of tubules and nodes may indicate more branching, which is characteristic of tumor vasculature compared to that of normal tissues (García‐Caballero et al. 2022). Alternatively, the reduction in the number of loops could mean less adhesion between cells, so they would not be able to maintain connections between each other by closing a gap, but this theory needs to be further investigated.

No changes were found in autophagy markers LC3 and p62, nor in BiP, in HUVEC treated with LEV 6.5. On the other hand, LEV T led to an increase in p62 levels, with no increment in LC3‐II detected. It has been frequently reported that an increase in LC3‐II occurs without changes in p62, since the former is converted more quickly than p62 degradation (Klionsky et al. 2021), so an increase in LC3‐II would also be expected. p62 can be accumulated by RAS signaling (Moscat and Diaz‐Meco 2009), an important protein for cell migration (Weber and Carroll 2021), a process that was increased by LEV T. This increase in p62 leads to the activation of Nuclear Factor kappa B (NFκB) (Moscat and Diaz‐Meco 2009), which may be related to the increase in permeability.

Activation of p‐eIF2α, part of the UPR, was found in HUVECs treated with LEV 6.5. This finding, considering the lack of change in viability of HUVECs, suggests that this activation of the UPR must have been mild. In fact, it is possible that the UPR was activated without ER stress. For example, VEGF activates p‐eIF2α without inducing dissociation between ATF6 and BiP (Karali et al. 2014). Greater activation of p‐eIF2α was also found in HUVEC treated with HeLa cell sEV (Lin et al. 2020), so it could be by the same mechanism, although still unknown. The Phosphorylation of eIF2α may or may not have been dependent on PERK. eIF2α can be phosphorylated by other kinases: PKR, HRI, and GCN2, depending on the type of stress (Donnelly et al. 2013). Activation of p‐eIF2α through this last kinase is crucial for keratinocyte migration (Miles et al. 2021). This may be related to the faster wound closure of HUVEC treated with LEV 6.5.

This study has limitations inherent to the model and methodology used. HUVEC were used instead of lymphatic vessels or breast tumors, since, as previously mentioned, NECs are very distinct from TECs (Maishi et al. 2019) and also from lymphatic endothelial cells (Podgrabinska et al. 2002). Additionally, there are several typical tumor microenvironment factors that could cause ER stress, and the transcriptional response, including the UPR, depends on the type of stimulus (BioProject PRJNA1053381 and unpublished GEOdataset GSE250335) (Ralph 2019), duration, and cell (Thews et al. 2024). The use of tunicamycin, a standard in the induction of ER stress, was a means of generalization.In conclusion, LEV from tumor cells adapted to acidosis cause slight activation of p‐eIF2α, without inducing autophagy or altering the metabolism of HUVECs (Figure 10). Here in this study, we observed that EV from tumor cells adapted to acidosis increase the migration of HUVECs, compared to control LEV, slightly decrease angiogenesis, inhibit adhesion between tumor cells and endothelial cells and increase endothelial permeability in response to treatment (Figure 10). LEV from tumor cells that underwent ER stress and adaptation to acidosis increase endothelial cell migration and permeability. However, we found no evidence that these LEV alter either tubulogenesis or tumor cell adhesion to endothelial cells. Additional studies are still needed to study the differential expression of the contents of these vesicles under such different conditions.

**Figure 10:**
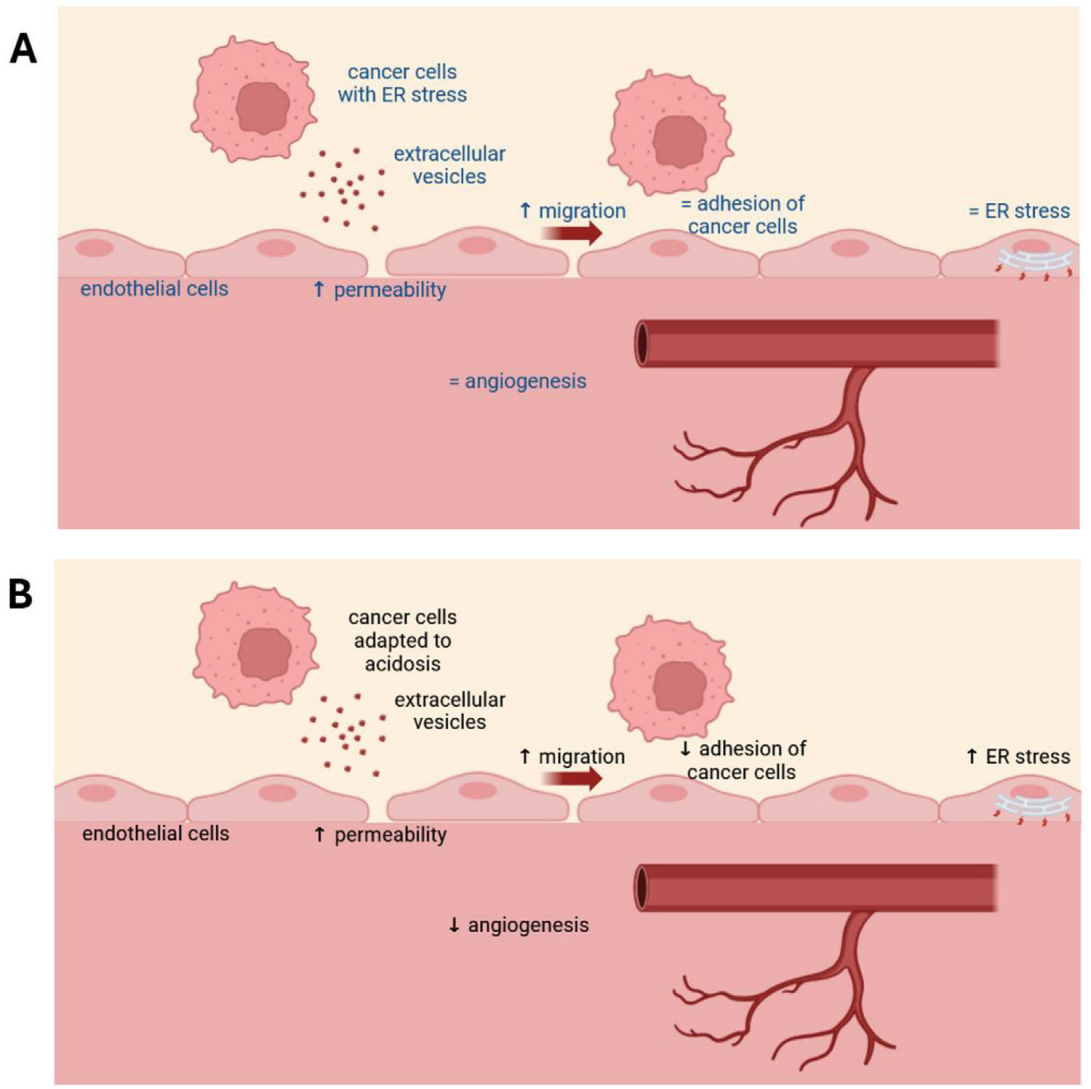
Illustrations of the findings: the effects of LEV T and LEV 6.5. When breast cancer cells experience ER stress, their large extracellular vesicles promote increased endothelial migration and endothelial permeability. When these cancer cells are adapted to acidosis, their extracellular vesicles increase permeability and migration, reduce angiogenesis and tumor cell adhesion to endothelia, and activate ER stress.

## 4 Methods

### 4.1 Tumor Cell Culture

The triple‐negative human breast cancer cell line MDA‐MB‐231 was cultured in tissue culture‐treated plates in Dulbecco's Modified Eagle Medium (DMEM) with 4.5 g/L glucose (Sigma‐Aldrich D5648) pH 7.2 supplemented with 5% fetal bovine serum (FBS) (Gibco 12657029) and 1% penicillin/streptomycin (100 U/mL penicillin, 100 µg/mL streptomycin) (Gibco 15140122), and maintained at 37°C and 5% CO_2_. For passage, cells were washed with phosphate‐buffered saline (PBS) and incubated with trypsin (Gibco 15090046) diluted 10x to 1x in versene. Trypsin was inactivated with medium containing FBS, and cells were centrifuged at 1200 rpm for 5 min. The pellet was resuspended in culture medium and distributed in new plates. Cells were expanded in a ratio of 1:2 to 1:6. The medium was changed every 2–3 days. To maintain cell lineage identity, cells were used until the 30th passage. To adapt tumor cells to acidosis, they were cultured for 30 days at pH 6.5, buffered with the addition of 4‐morpholinoethanesulfonic acid (MES) (20 mM) and HCl (Michl et al. 2019). As a control, cells cultured at pH 7.2 buffered with the addition of 20 mM 4‐2‐hydroxyethyl piperazine‐1‐ethanesulfonic acid (HEPES) were used.

### 4.2 Endothelial Cell Culture

Human umbilical cord vein endothelial cells (HUVEC) were purchased from Lonza and thawed on tissue culture‐treated plates pre‐coated with 0.1% gelatin (Sigma‐Aldrich G9136) and allowed to adhere for 4 h before changing the medium. HUVECs were cultured in Endothelial Cell Growth Basal Medium‐2 (EBM‐2) (Lonza CC‐3156) with EGMTM‐2 SingleQuotsTM Supplements (Lonza CC‐4176), 37°C, 5% CO_2_, and 100% humidity. For subculture, cells were washed with versene and incubated with trypsin. Trypsin was inactivated with FBS‐containing medium, and cells were centrifuged at 800 rpm for 5 min. The pellet was resuspended and distributed into new plates previously coated with gelatin. Cells were expanded in a 1:2 or 1:3 ratio. The medium was changed every 2–3 days. Due to cellular senescence, HUVECs were cultured until passage 8.

### 4.3 Western Blot

The RIPA (Radio‐Immunoprecipitation Assay) lysis buffer, Laemmli, running, transfer, and Tris‐buffered saline with Tween 20 (TBST) and Ponceau S staining solution were prepared according to the BIO‐RAD protocol (BIO‐RAD n.d.). Cells were lysed with RIPA buffer on ice for 10 min and mechanically disrupted with a cell scraper. The sample was centrifuged at 15,000 g and 4°C for 10 min. Protein in the supernatant was quantified by PierceTM BCA protein assay following the manufacturer's instructions (Thermo Scientific A55864). Samples were concentration normalized with RIPA, 4X concentrated Laemmli sample buffer was added, and boiled at 95°C for 5 min. 16 µg of protein (cells) or 4 µg of protein (EVs) were loaded per well. Samples were run on a 4%–20% or 12% polyacrylamide gel (EVs) at 100 V and transferred to a 0.22 µm pore nitrocellulose membrane at 18 V, 4°C for 16 h. The membranes were blocked with 5% bovine serum albumin (for phosphorylated proteins) or skim milk powder in TBST for 1 h at room temperature with rocking. Antibodies were diluted 1:1000 in blocking buffer. Membranes were incubated in primary antibody at 4°C overnight, washed three times, and incubated in secondary antibody at room temperature for 1 h. The antibodies used were eIF2α (CST, 9722), p‐eIF2α (CST, 3398), XBP1s (CST, 40435), BiP (CST, 3177), p62 (CST, 8025), LC3B (CST, 12741), annexin A1 (Invitrogen, MA541551), GM130 (CST 12480), β‐actin (Sigma, A5316), followed by incubation with HRP‐conjugated secondary antibodies: anti‐rabbit IgG (CST, 7074) or anti‐mouse IgG (CST, 7076). Membranes were revealed using Clarity Western ECL Substrate (BIO‐RAD, 1705061) and documented on Amersham Imager 600 (GE Healthcare Life Sciences). Before and after antibody incubation, membranes were washed in TBST for 5 min at room temperature three times. When necessary, antibody removal was performed with Restore Western Blot Stripping Buffer (ThermoFisher, 21059) by rocking at 37°C for 15 min. The membrane was washed, revealed, blocked again, incubated with secondary antibody, and revealed to verify primary antibody removal.

### 4.4 Isolation of Extracellular Vesicles

MDA‐MB‐231 cells were seeded in 150 mm tissue culture‐treated Petri dishes at 70% confluence and treated with tunicamycin (Sigma‐Aldrich, T7765) or the same concentration of dimethyl sulfoxide (DMSO) vehicle for 24 h. The cells were then washed 3 times with Hank's balanced salt solution (Sigma‐Aldrich H4891) to avoid contamination of EVs from the FBS and incubated in serum‐free medium for 24 h. In the case of acidosis‐adapted cells, after reaching confluence, the cells were washed 3 times and incubated in serum‐free medium. The conditioned medium was centrifuged in 50 mL conical tubes at 2,000 g for 10 min at 4°C in the fixed‐angle rotor F‐34‐6‐38 of the Eppendorf centrifuge model 5804R. The supernatant was transferred with a serological pipette to another tube and centrifuged at 15,000 g for 30 min. The pellets were resuspended in 200 µL of filtered PBS (0.22 µm pores) in each tube. To reduce contaminating free proteins (Théry et al. 2006; Webber and Clayton 2013), the pellets (obtained from 12 150 mm culture plates) were pooled in microtubes, centrifuged at the same parameters as the previous centrifugation, and resuspended in 30–50 µL of filtered PBS. For protein quantification, 5 µL of the EV sample was diluted to 25 µL with 5 µL of 5x RIPA and the remainder with ultrapure water. After quantification, aliquots were frozen at ‐80°C. Protein quantification was used to estimate the EV concentration of the samples, and thus to apply a volume of EVs in suspension corresponding to the same amount of EVs for treatment of HUVECs.

### 4.5 Nanoparticle Tracking Analysis (NTA)

The number of particles and the size of EVs were measured by a Nanoparticle Tracking Analysis (NTA) equipment (Nanosight LM20, coupled to a charge‐coupled device (CCD) camera and a laser that emits a 60 mW beam at a wavelength of 532 nm). The amount of EV corresponding to 1 µg of protein was diluted to 300 µL with filtered PBS. The sample was injected, and five videos of 30s were collected to determine the total particle concentration and size (hydrodynamic radius). The analysis was kindly performed by Dr. Amanda Ikegami, a student of Prof. Dr. Tiago Góss dos Santos (A.C. Camargo).

### 4.6 Immunogold Assay of Extracellular Vesicles by Transmission Electron Microscopy

The protocol used was modified from (Capomaccio et al. 2019). A volume of EV suspended in PBS corresponding to 4 µg of protein was diluted in PBS to 20 µL. This volume was adsorbed on a nickel grid coated with formvar and carbon (Koch Electron Microscopy LTDA. FCF200‐400 MESH‐NI‐50) for 1 h at room temperature. Excess liquid was removed with filter paper, and the sample was blocked in 10% donkey serum in PBS for 10 min. The grids were washed with 1% bovine serum albumin (BSA) in filtered PBS (0.22 µm pores) in droplets twice for 2 min. The grids were incubated in 20 µL of anti‐annexin A1 (Thermo MA5‐41551) diluted 1:10 in PBS 1% BSA in a humid chamber at room temperature overnight. Then, the grids were washed in droplets 8 times in PBS for 2 min, and incubated in 20 µL of donkey anti‐mouse IgG conjugated to 18 nm gold particles (Jackson ImmunoResearch 715‐215‐150) diluted 1:10 in PBS 1% BSA for 2 h at room temperature in a humid chamber. The grids were washed 8 times for 2 min, fixed in 2.5% glutaraldehyde for 10 min, washed 3 times for 2 min, and dried for 1 h at room temperature. Counterstaining was done with 2% uranyl acetate for 5 min. Transmission electron microscopy micrographs were captured with a Tecnai 20 microscope. Vesicles were found using 22,000x magnification.

### 4.7 Transmission Electron Microscopy (TEM) of Cells

Cells were prepared as detailed in (Silva et al. 2016). Cells seeded in 60 mm tissue culture Petri dishes in confluency trypsinized, pelleted, resuspended in PBS, centrifuged and ressuspended in 2.5% glutaraldehyde in 0.1 M sodium cacodylate buffer (pH 7.2) and stored in 4°C. The cells were postfixed in 1% osmium tetroxide for 2 h, washed in distilled water, stained in 0.5% uranyl acetate, rinsed and dehydrated in graded ethanol. After immersion in propylene oxide, samples were embedded in epoxy resin (Spurr, Electron Microscopy Sciences, EMS, Hatfield PA, USA) and polymerized for 42 h at 75°C. Sections (0.5 µm thick) were stained in 1.0% toluidine blue in 1.0% aqueous sodium borate for light microscopic examination. Ultrathin sections were stained with lead citrate and uranyl acetate and examined in a JEOL 1010 transmission electron microscope (Jeol Inc., Peabody, MA, USA) at 80 kV.

### 4.8 High‐Performance Liquid Chromatography (HPLC)

Tunicamycin (Sigma‐Aldrich T7765) solubilized in DMSO was diluted in filtered methanol (0.22 µm) to 100 µg/mL. 2 µL of LEV was diluted in 48 µL of lysis buffer 250 nM Tris‐HCl, pH 7.4; 2.5% SDS (Mukhopadhya et al. 2023) and vortexed. The volume was brought to 1 mL with filtered methanol, and the tube was centrifuged at 3200 rpm for 5 min at 20°C. The supernatant was collected and filtered through a 0.22 µm filter. Tunicamycin and LEV were run on a C‐18 column using a mobile phase of 45:55 v/v acetonitrile: 1% v/v acetic acid, 1 mL/min for 60 min in isocratic elution and detection by UV 259 nm in HPLC (Tsvetanova and Price 2001). The analyses were kindly performed by Anayara Ristow, a student of Prof. Dr. Daniel Pecoraro Demarque (Faculty of Pharmaceutical Sciences, University of São Paulo), in collaboration.

### 4.9 MTT Assay

MDA‐MB‐231 cells were seeded at 2.82 × 10^4^ cells in 200 µL per well in a 96‐well plate. On the following day, the cells were incubated for 24 h with increasing concentrations of Tm. MTT assay was performed at this point or after incubating them in serum‐free media for 24 h following the referred treatment with Tm.

For MTT assay with HUVECs, the cells were seeded at 1.5 × 10^4^ cells in 200 µL per well in a 96‐well plate and left to adhere for 1 day. The cells were then incubated with EVs at 0.5 µg/mL for 24 h. The treatment with 0.5 mg/mL MTT (3‐(4,5‐dimethylthiazol‐2‐yl)‐2,5‐diphenyltetrazolium bromide) was applied for 3 h. The medium was discarded, and the formazan crystals were dissolved in 100 µL of DMSO. Absorbance at 540 nm was measured using a spectrophotometer (BioTek, Winooski, Vermont, USA).

### 4.10 Adhesion Assay

HUVECs were seeded at 1 × 10^5^ cells in 500 µL per well in a 24‐well plate with 13 mm glass coverslips sterilized by autoclaving at the bottom and left to adhere for 1 day. Once confluence was reached, the cells were incubated with EVs at 0.5 µg/mL for 5 h. The medium was removed, and the cells were incubated with 1 × 10^5^ MDA‐MB‐231‐RFP (expressing red fluorescent protein) per well, suspended in DMEM with 5% FBS for 30 min. The cells were washed once with serum‐free DMEM to remove non‐adherent cells, fixed with 4% paraformaldehyde in PBS for 10 min, and washed three times with PBS. VCAM‐1 was stained by immunofluorescence with CST, 39036 1:400, and anti‐rabbit 488 1:200 (Invitrogen, A11034), and the nuclei were stained with ProLongTM Diamond Antifade Mountant with DAPI (InvitrogenTM, P36962). Images were captured using a ZEISS Axio Observer 7 fluorescence microscope at 20x magnification. The nuclei, the adhered MDA‐MB‐231 cells, and VCAM‐1‐positive cells were counted using ImageJ.

### 4.11 Wound Healing Assay

Cell migration was evaluated using a wound healing/scratch assay. 1.5 × 10^4^ HUVECs in 200 µL per well were seeded in a 96‐well plate and left to adhere for 1 day to reach confluence. The cells were then incubated with EVs at 0.5 µg/mL for 24 h. The medium was replaced with medium containing 5 µg/mL mitomycin. A scratch was made 2 h later using the Incucyte Woundmaker Tool (Sartorius, 4563), which removes cells in a standardized and linear manner, and the medium was replaced with fresh medium containing the same EV treatment. Images at 0 h and 16 h after the scratch were captured using the BioTek Lionheart FX Automated Microscope (Agilent) at 4x magnification with phase contrast in the well medium. Using Gene5 software version 3.8, the optical meniscus effect was eliminated with the “background flattening” tool. The scratch area was manually delimited and measured using ImageJ, and the percentage of the wound area at 16 h compared to 0 h was calculated. No automatic wound area delimitation was used because the cells were migrating scattered since their proliferation was inhibited by mitomycin.

### 4.12 Tube Formation Assay

Angiogenesis was evaluated by tube formation assay. The μ‐Slide Angiogenesis (IBIDI 81506) was filled with 10.4 µL of Matrigel (Corning 356231) kept on ice. After achieving a flat surface of Matrigel, the μ‐Slide was placed in a Petri dish with wet paper inside the incubator and left to gel for 30 min. HUVECs were trypsinized, and 2 × 10^4^ cells per well, with 5 wells per condition, were prepared in 1.5 mL in a 2 mL conical‐bottom microtube, centrifuged, and resuspended in medium with EVs at 0.5 µg/mL, 50 µL per well. The cells were seeded in the wells and incubated for 16 h. Images were taken with phase contrast at 4x magnification using an Olympus inverted microscope with an attached camera (Moticam, Motic A1). Loops and nodes were counted, and tube length was measured with ImageJ using the Angiogenesis Analyzer plugin, available at http://image.bio.methods.free.fr/ImageJ/?Angiogenesis‐Analyzer‐for‐ImageJ&lang=en.

### 4.13 Immunofluorescence

MDA‐MB‐231 cells were seeded in a 24‐well plate at 5 × 10^4^ cells per well with autoclaved 13 mm glass coverslips at the bottom, and allowed to adhere for 1 day. The cells were fixed with 4% paraformaldehyde in PBS for 10 min and washed three times with PBS. Permeabilization and blocking were performed with 3% goat serum, 0.3% Triton X‐100, and 0.05% Tween 20 in PBS for 1 h at room temperature. The coverslips were incubated with anti‐annexin A1 (Invitrogen, MA541551) diluted 1:100 in the same buffer at 4°C overnight. The coverslips were washed three times and incubated with anti‐mouse IgG conjugated to Alexa Fluor 488 (Invitrogen, A21202) at 1:400 in blocking buffer. The coverslips were washed in PBS and mounted on slides with ProLong Diamond Antifade Mountant with DAPI (Invitrogen™ P36966). Images were captured using a Nikon Eclipse E600 microscope with a Nikon Intensilight C‐HGFI lamp and an Olympus DP72 camera, using a 40x objective.

HUVECs were seeded in a 24‐well plate at 1 × 10^5^ cells in 500 µL per well with autoclaved 13 mm glass coverslips at the bottom, and allowed to adhere for 1 day. Once confluent, the cells were incubated with EVs at 0.5 µg/mL for 24 h. The cells were fixed, permeabilized, and blocked using the same method. The coverslips were incubated with anti‐VE‐cadherin (Santa Cruz, sc‐9989) diluted 1:50 in the same buffer at 4°C overnight. The coverslips were washed three times and incubated with anti‐mouse IgG 488 (Invitrogen, A11001). The coverslips were washed in PBS and mounted on slides with the same mounting medium. Images were captured on a ZEISS Axio Observer 7 microscope with 20x and 63x magnifications, the latter using the z‐stack mode at 0.24 µm intervals.

### 4.14 Permeability Assay in Organ‐on‐a‐Chip

Microfluidic organ‐on‐a‐chip devices known as TANDEM were used to evaluate the permeability of endothelial cells in response to EVs. TANDEM devices were microfabricated as previously described (Mohan and Young 2021). The device design consisted of three parallel channels separated by phaseguides (Figure 11). When a collagen gel was injected into the middle channel, the phaseguides prevented the gel from advancing into the lateral channels, thereby creating an interface between the gel and the liquid medium delivered into the two side channels.

**Figure 11:**
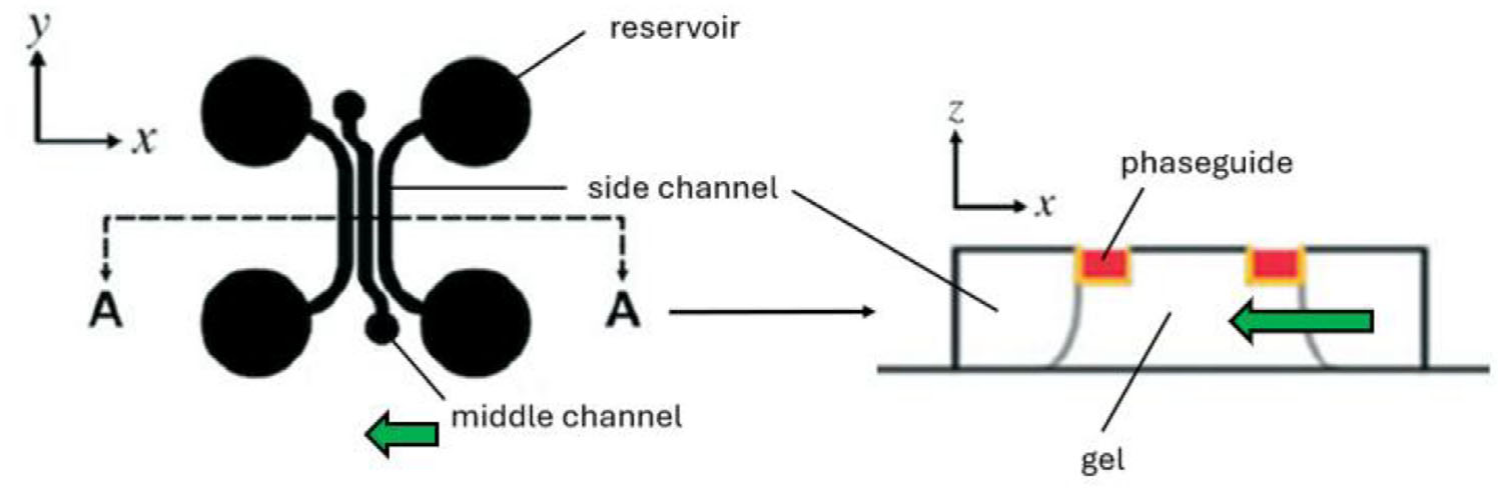
Representative diagram of the modified microfluidic device design from (Mohan and Young 2021). Each unit consisted of a middle channel and two lateral side channels (left). Each end of the lateral side channels is connected to a media reservoir. In a cross‐section view (right), two phaseguides prevent the propagation of liquid from the middle channel when it is injected. The arrows indicate the direction in which fluorescein isothiocyanate‐dextran (FITC‐dextran) diffuses in the assay. Image adapted from Mohan (Mohan and Young 2021).

### 4.15 Type I Collagen Gel Preparation and Experimental Protocol

Type I collagen gel (Corning CLS354249) at 5.5 mg/mL was prepared by mixing with DMEM, FBS, and penicillin/streptomycin to achieve a final concentration of 1x DMEM, 2% FBS (as in EGM2), and 1% penicillin/streptomycin, with a final pH of 7.4, ideal for collagen polymerization. The gel was injected into the middle channel and allowed to polymerize for 30 min in a pre‐wet plate in the incubator. Then, 10 µL of HUVEC suspension (6–8 × 10^6^ cells/mL) were injected into one of the lateral channels, and medium was injected into the other lateral channel. The chip was placed on a slanted plate in the incubator for 20 min. The lateral channel with HUVECs had volume replacement of 20 µL twice to remove non‐adherent cells. On the following day, FITC‐dextran 70 kDa (Sigma‐Aldrich, 46945) was dissolved in EGM2 to a final concentration of 20 mg/mL. Extracellular vesicles (EVs) at 0.5 µg/mL or tumor necrosis factor‐alpha (TNF‐α) at 100 ng/mL were diluted in medium containing FITC‐dextran. The medium was removed from the reservoirs, and 13 µL of this treatment medium was added and homogenized. Images were taken from 0 to 60 min at 10‐min intervals using the EVOS FL Auto (ThermoFisher Life Technologies) with 2x magnification.

### 4.16 Immunofluorescence of VE‐Cadherin in HUVECs on Organ‐on‐a‐Chip

The day after HUVEC seeding, the cells were washed with warm PBS containing calcium and magnesium (Sigma‐Aldrich, D8662‐500ML), fixed with 100% methanol at ‐20°C for 10 min, then brought to room temperature and washed three times with PBS. The cells were permeabilized with PBS 0.1% Triton X‐100 for 5 min, washed three times, and blocked with PBS 1% BSA for 30 min. The sample was incubated with anti‐VE‐cadherin (Abcam, ab33168) at 3 µg/mL in blocking buffer for 4 h at room temperature. The channel was washed three times, incubated with Alexa Fluor 568 anti‐goat rabbit (Thermo Fisher Scientific, Invitrogen, A‐11011) at 1:100 in blocking buffer for 1 h at room temperature, and washed three times. For nuclear staining, the cells were incubated with Hoechst (Thermo Fisher Scientific, Invitrogen, H3570) at 1:1000 in PBS for 5 min and washed three times (Mohan and Young 2021). The HUVEC layer was photographed with the Leica Stellaris 5 LightSheet confocal microscope at the Centre for Research and Applications in Fluidic Technologies (CRAFT), using a 10x objective in a z‐stack of 70–80 steps. Z‐projections were made using Fiji software.

## 5 Statistical Analysis

Initially, the data from each group were tested for normality using the Shapiro‐Wilk test. Significant differences between the means were then assessed using one‐way ANOVA or Kruskal‐Wallis tests for normal and non‐normal distributions, respectively. If significant differences were confirmed between any group, Fisher's Least Significant Difference or unprotected Dunn's test was performed for normal and non‐normal distributions, respectively (GraphPad 2024; Rothman 1990).

## Author Contributions

**Ana Sayuri Yamagata**: data curation, formal analysis, investigation, methodology, visualization, Writing–original draft. **Ramon H. G. Teles and Nicolas J. Villarinho**: investigation, review, and editing. **Ana Carolina Lima Ralph and Nicolly C. dos Santos**: investigation, formal analysis. **Kevin Perera**: investigation, methodology. **Gisela R. Terçarioli, Amanda Ikegami, Anayara Ristow and Jeremy Newton**: investigation. **Luiz E. da Silva. and Daniel Pecoraro Demarque**: methodology. **Ruy G. Jaeger**: funding acquisition, supervision. **Edmond W. K. Young**: conceptualization, project administration, supervision. **Vanessa Morais Freitas**: conceptualization, funding acquisition, project administration, supervision, writing – review & editing.

## Funding

This work was supported by the São Paulo Research Foundation under the grant numbers 2020/15696‐4 to V.M.F. and 2020/15751‐5 and 2023/05006‐9 (A.S.Y.scholarships).

## Conflicts of Interest

The authors declare no conflicts of interest.

## Supporting information




**Supporting File 1**: boc70085‐sup‐0001‐Fig S1A.JPG.

**Supporting File 2**: boc70085‐sup‐0002‐Fig S1B.JPG.

**Supporting File 3**: boc70085‐sup‐0003‐Fig S1C.JPG.

**Supporting File 4**: boc70085‐sup‐0004‐Fig S1B.JPG.

**Supporting File 5**: boc70085‐sup‐0001‐Fig S1E.JPG.

## Data Availability

All authors confirm that the data supporting the findings of this study are available within the article. The datasets used and/or analyzed during the current study are available from the corresponding author upon reasonable request.
